# New Virtual Reality Educational Tool for Evaluating Dental Mirror Technique Skills: A Pilot Study

**DOI:** 10.3390/dj13120566

**Published:** 2025-12-01

**Authors:** Rei Nagasawa, Takumi Sato, Yuto Isogai, Yui Yamada, Takashi Imamura, Noritaka Fujii

**Affiliations:** 1 Division of Dental Clinical Education, Faculty of Dentistry, Graduate School of Medical and Dental Sciences, Niigata University, Niigata 951-8514, Japan; nrei@dent.niigata-u.ac.jp (R.N.); norisuke@dent.niigata-u.ac.jp (N.F.); 2 General Dentistry and Clinical Education Unit, Niigata University Medical and Dental Hospital, Niigata 951-8520, Japan; 3 Division of Periodontology, Department of Oral Biological Science, Faculty of Dentistry, Graduate School of Medical and Dental Sciences, Niigata University, Niigata 951-8514, Japan; 4 Smart Information Systems Program, Faculty of Engineering, Niigata University, Niigata 950-2181, Japanima@eng.niigata-u.ac.jp (T.I.)

**Keywords:** dental education, virtual reality, dental mirror, mirror technique, dental simulation and skills evaluation, digital education

## Abstract

**Background/Objectives:** Appropriate use of a dental mirror (MT) is essential for dental treatment. However, because the mirror image differs depending on the operator’s position, there are few effective tools available to train dental students and inexperienced dentists. To address this issue, a new virtual reality (VR) system was developed. **Methods:** Eighty-seven participants, including instructor dentists, graduate students, trainee dentists, and dental students from Niigata University, participated in the experiment. The participants manipulated the virtual dental mirror (DM) connected to three-dimensional (3D) control devices while wearing a head-mounted display (HMD) to simulate an intraoral examination and caries removal. The distance between the center of the DM and the caries, area ratio of the caries on the DM, ellipticity of the caries image, and manipulation time were measured and compared between groups. **Results:** The mean distance between the centers was 1.18 mm for instructor dentists and 1.56 mm for the students (*p* = 0.003; effect size, 0.48). Instructor dentists demonstrated significantly superior performance in all evaluation categories compared to the other groups. This suggests that the level of clinical experience affected the results. **Conclusions:** The newly developed VR system can quantitatively and objectively evaluate DM manipulation. Differences in the operator’s clinical experience led to variations in the techniques used for mirror image confirmation and DM manipulation. This VR system enables quantitative feedback by outputting images from the HMD to a monitor. Therefore, it has the potential to evolve into an unprecedented training tool for dental students or trainees.

## 1. Introduction

In recent years, numerous tools and systems have been developed for dental clinical training, including traditional mannequin training and the use of various virtual reality (VR) technologies [1,2], such as the Simodont dental trainer (Moog, Nieuw-Vennep, The Netherlands) [3,4,5] and other devices [6,7,8,9]. These devices have enabled hands-on treatment and training in a variety of subjects in the dental curriculum [10,11,12,13,14,15,16,17,18,19,20]. Simulators have gained attention, owing to their advantages in allowing safe and repeated training [21]. Previous studies on dental treatment using simulators revealed that appropriate feedback was considered crucial for effective learning [22,23,24].

However, proper handling of dental mirrors (hereafter referred to as the Mirror Technique [MT]) is considered an essential skill for dentists performing procedures in confined spaces. Since the mirror image changes depending on the practitioner’s position, even with the latest devices, the best mode for MT education has yet to be established. Our previous research indicated that MT proficiency is closely related to the practitioners’ clinical experience [25,26]. Recently, VR technology has been applied to dental clinical education, demonstrating usefulness [5,8,11,12,15,16,18,23]. Therefore, we hypothesized that clinical experience significantly affects VR-based MT performance metrics and accordingly developed a new VR system to enable instructors to share mirror images and dental mirror manipulations performed by trainees. This study aimed to establish a method for quantitatively and objectively evaluating MT skills and to develop an unprecedented MT training approach.

## 2. Materials and Methods

This study was approved by the Ethics Committee of Niigata University Hospital (approval number 2018-0332). All procedures were conducted in accordance with the ethical guidelines and principles put forth in the Declaration of Helsinki.

### 2.1. Overview of Dental Mirror Virtual Reality System

#### 2.1.1. Function

Users wearing head-mounted displays (HMDs) can examine a patient’s intraoral space reproduced in a VR environment using a device connected to three-dimensional (3D) controls. The HMD acquires the user’s head position data and dynamically adjusts the presented images as in actual clinical practice. The virtual dental mirror (DM) has reflective properties similar to those of a real dental mirror and shows an image that reflects the direction of the user’s gaze based on the head position data acquired. The image projected onto the DM updates based on its reflection, position, and orientation, as well as the user’s head position and gaze direction. The visual field information presented to the user and the reflected image can also be displayed on a monitor.

#### 2.1.2. Construction

**Figure 1:**
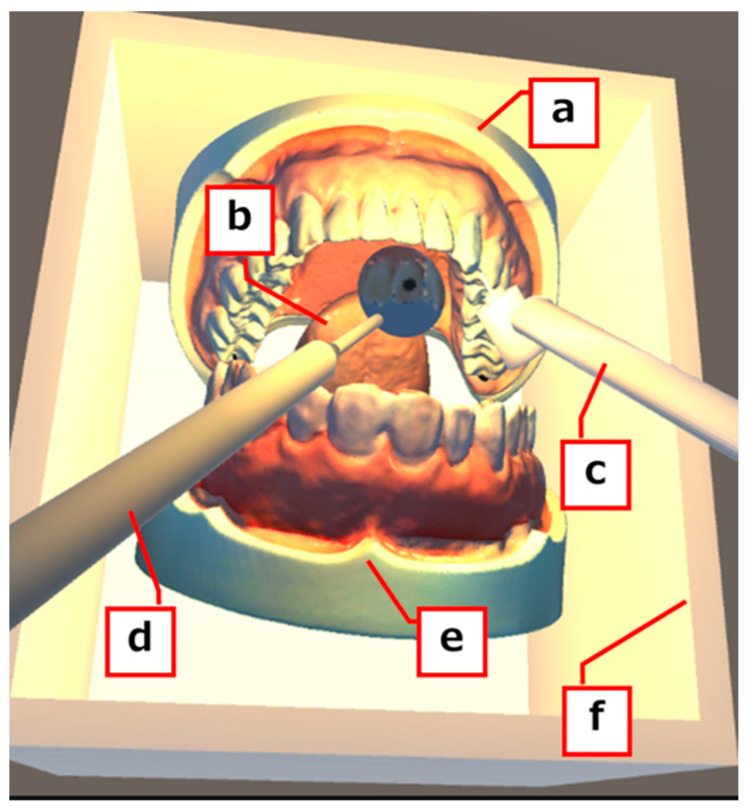
Appearance of the virtual reality system. (a) Mandibular model, (b) tongue, (c) air turbine handpiece, (d) dental mirror, (e) maxilla model, (f) frame.

The system constructed in this study is illustrated in Figure 1.

**Figure 2:**
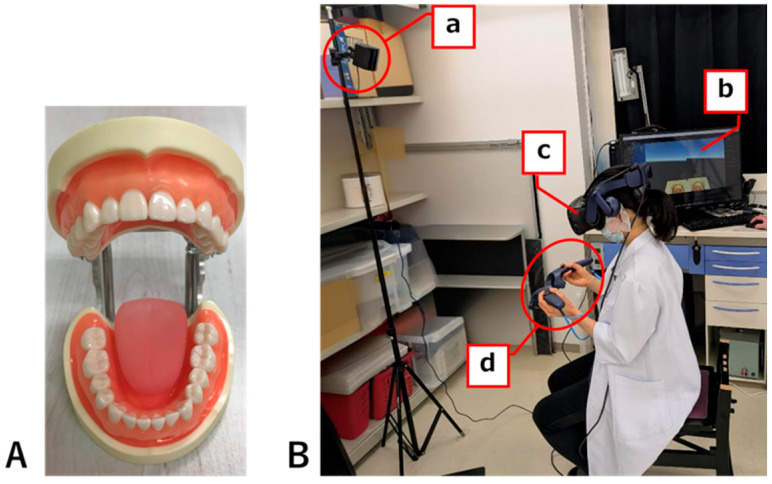
Experimental instruments and environment. (**A**) Dentition model and model tongue (NISSIN) imported into the VR system. (**B**) Experimental setup used in this study: (a) base station, (b) PC, (c) head-mounted display, (d) 3D controllers (VIVE Pro2).

A fundamental VR environment was built using the Unity game engine (Unity Technologies, San Francisco, CA, USA). A dental mirror and an air-turbine handpiece (TH) placed within the VR environment were created using Blender (Blender Foundation, Amsterdam, The Netherlands), an integrated 3D computer graphics production environment. The dentition and tongue models (NISSIN, Kyoto, Japan) were scanned using a 3D scanner (Seal Lite 3D; 3DMakerpro, Shenzhen, China) to create 3D data, which were then used to replace the original physical models with virtual ones (Figure 2A).

Instrument handling within the VR space was performed using 3D controllers (VIVE Pro2, HTC, New Taipei City, Chinese Taipei) linked to the DM and TH. The PC had the following specifications: CPU, Intel Core i7-12700F; memory, DDR4-3200 32 GB; and GPU, Nvidia GeForce RTX3060 GDDR6 12 GB.

#### 2.1.3. Configuration

The following settings were implemented to display the reflected images in the DM, corresponding to the participant’s head position and line of sight: multiple cameras representing the user’s viewpoints were setup in Unity. A mirror-image camera was positioned symmetrically relative to the user’s viewpoint camera, using the DM surface as the axis of symmetry. The mirror image of the camera was adjusted to fit the mirror surface size within the field of view, and the objects between the mirror surface and the mirror image camera were ignored. A DM with a diameter of 20 mm and a black caries lesion (BC) with a diameter of 3 mm, each a perfect circle, were created to match the actual measurements of the scanned model.

To prioritize mirror-image generation, the VR configuration in this study was simplified in two ways. First, as actual oral examinations involve compression of the lips and cheek mucosa, and instrument manipulation is restricted. However, modeling and implementing these elastic tissues would require complex behavior calculations and costly computation, consequently causing slow processing speed. Therefore, oral caries were alternatively recreated by installing a frame surrounding the dentition model. Second, we simplified the setting of object interactions within the VR environment. The implementation of collisions between objects in Unity requires a definition of their mass and physical properties. Similarly to those in the real environment, objects in a VR environment can be treated as rigid bodies by setting gravity-related phenomena to reproduce object-to-object contact. However, setting the mass is required, and objects that are not being manipulated cannot remain in the VR space. Consequently, rigid-body settings were not used in this study (Figure 2B).

### 2.2. Participants

A total of 87 individuals without any disability participated in this study. This included 25 instructor dentists (ID) with over 9 years of clinical experience (15 men, 10 women, age range 33–60 years, average age 43.8 ± 8.3 years) and 17 trainee dentists (TD) (9 men, 8 women, age range 24–32 years, average age 25.2 ± 1.9 years) affiliated with Niigata University Hospital and 24 graduate students (GS) in their first through fourth years (12 men, 12 women, age range 25–34 years, average age 27.9 ± 2.1 years), and 21 students (ST) in their sixth year (8 men, 13 women, age range 23–27 years, 23.8 ± 1.1 years) from the Department of Dentistry, Niigata University School of Dentistry. The experiment also involved an experimenter who supervised the participants and assistants.

### 2.3. Experimental Procedure

**Figure 3:**
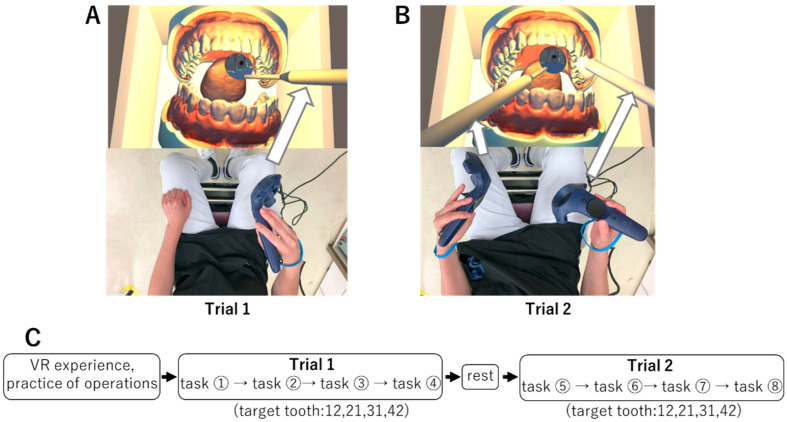
Experimental protocol and trials. (**A**) Trial 1. Holding the DM with the right hand. (**B**) Trial 2. Holding the TH with the right hand and the DM with the left hand. (**C**) Experimental protocol. Four tasks were performed in both Trial 1 and Trial 2. Target teeth were randomly selected from 12, 21, 31, and 42.

Each participant practiced handling the VR device for five minutes to get accustomed to its manipulation. Subsequently, they conduct two types of trial handling: DM and TH. In Trial 1, the task involved manipulating a controller linked to the DM using the right hand to check the entire BC. In Trial 2, the operator wore a VR controller linked to the TH in the right hand and the DM in the left hand. The DM was manipulated to confirm the removal of the BC without performing any cutting with TH (Figure 3A,B). The participant was instructed to begin the manipulation upon the examiner’s sign, stop the manipulation once the position of the DM and the reflection of the BC mirror image were judged to be appropriate (the appropriate mirror position), and then notify the examiner. Each trial consisted of four tasks (tasks ①–⑧) randomly targeting the maxillary right lateral incisor (12), maxillary left central incisor (21), mandibular left central incisor (31), and mandibular right lateral incisor (42). Because complex technical settings are required to obtain an appropriate mirror image under all conditions, we initially focused on the anterior teeth, which provide the simplest conditions, with the goal of applying the technique to the molar area in the future. In Trial 1, the criteria for determining the appropriate mirror position were explained as two conditions: “The entire mirror must be visible” and “The mirror must not penetrate the tongue, mucosa, or teeth.” In Trial 2, an additional condition was added: “The tip of the TH must be within the virtual box and must not overlap the mirror image of the BC” (Figure 3C). The examiner recorded the time taken by the participant to stop the manipulation (manipulation time) and took a screenshot to determine the appropriate mirror position on the PC monitor. A two-minute break was scheduled between Trials 1 and 2. In the VR environment, the dentition model was positioned with its occlusal plane perpendicular to the ground, and the participants performed the task in the 12 o’clock practical position.

### 2.4. Questionnaire

**Figure 4:**
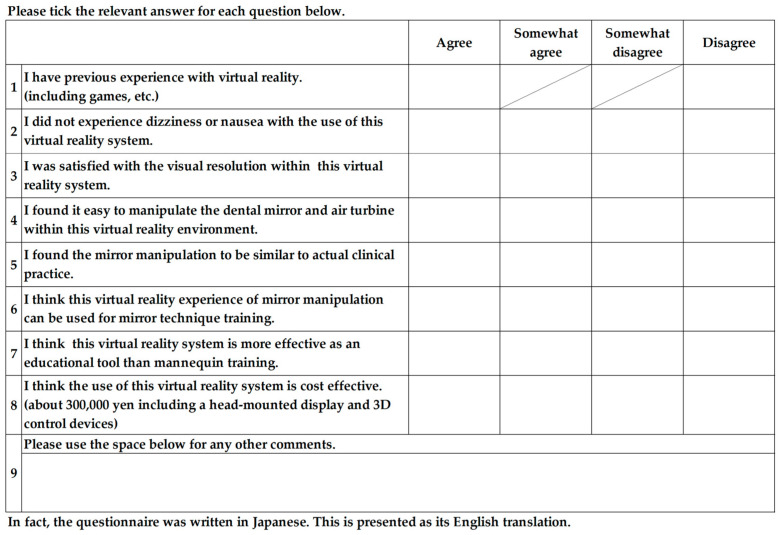
Questionnaire used for the experiment.

A questionnaire survey was created based on that used by Yamazaki et al. [27] and comprised nine questions regarding VR and MT in dental treatment (Figure 4).

### 2.5. Data Analysis

ImageJ software (Ver. 1.54g, National Institutes of Health, Bethesda, MD, USA) was used to calculate the major and minor axes of the elliptical DM and BC, as well as the respective areas, on the screenshot for each task. The inclination angles of the DM surface (θ) were also obtained. MATLAB R2025a (Ver. 25.1, MathWorks, Natick, MA, USA) was used for data calculation.

#### 2.5.1. Distance Between the Center of the DM and BC Mirror Image

Units of distance were converted from pixels to millimeters because the magnification ratio differed for each image, and the lengths of the centers of the DM and BC mirror images were calculated (distance between the centers) (Supplementary Figure S1A–D).

#### 2.5.2. Relative Ratio of BC Mirror Image on the DM Surface

The areas of the DM surface and the BC mirror image and the relative ratio of the BC mirror image on the DM surface were calculated (Supplementary Figure S1E).

#### 2.5.3. Ellipticity of BC Mirror Image on the DM

The ellipticity of the BC mirror image was calculated from the major and minor axes of ellipticity displayed in the DM (Supplementary Figure S1F).

#### 2.5.4. Manipulation Time

The manipulation time for each participant was recorded by an experimental assistant.

### 2.6. Analysis

The Kolmogorov–Smirnov test did not indicate normality for any data. Therefore, the Friedman test, a nonparametric test, was used to confirm differences in variability among groups. Subsequently, multiple comparisons were conducted to verify whether differences existed between individual groups. Friedman tests were performed with the distance between centers, relative ratio of BC, and ellipticity of BC as dependent variables. Comparisons between ID, GS, TD, and ST groups and between Trials 1 and 2 were performed using Bonferroni multiple comparisons. The Friedman test was also used for the manipulation time, and Bonferroni multiple comparisons were used to verify differences between ID, GS, TD, and ST groups. All statistical analyses were performed using the Bell Curve for Excel (Ver. 4.09, Social Survey Research Information Co., Tokyo, Japan) with a significance level of *p* < 0.05, and the effect size (d) was calculated for all results.

## 3. Results

### 3.1. Participant Characteristics and Questionnaire Survey

Almost all participants were right-handed, with the exception of three left-handed students. However, all participants used their right hand to handle dental instruments at the clinical site. The years of clinical experience ranged from 9 to 36 years for ID, 1 to 4 years for GS, approximately 3 months of clinical training for TD, and approximately 6 months since the start of clinical practice for ST (Table 1). Although the three participants in the ST group were left-handed, clinical practical training was uniformly conducted regardless of the dominant hand; moreover, their clinical experience was insufficient to significantly affect their performance. Therefore, this parameter was considered to have a minimal effect on the results. The TD, GS, and ID groups included participants specializing in conservative dentistry, prosthodontics, oral surgery, pediatric dentistry, and orthodontics.

### 3.2. Distance Between Centers

**Figure 5:**
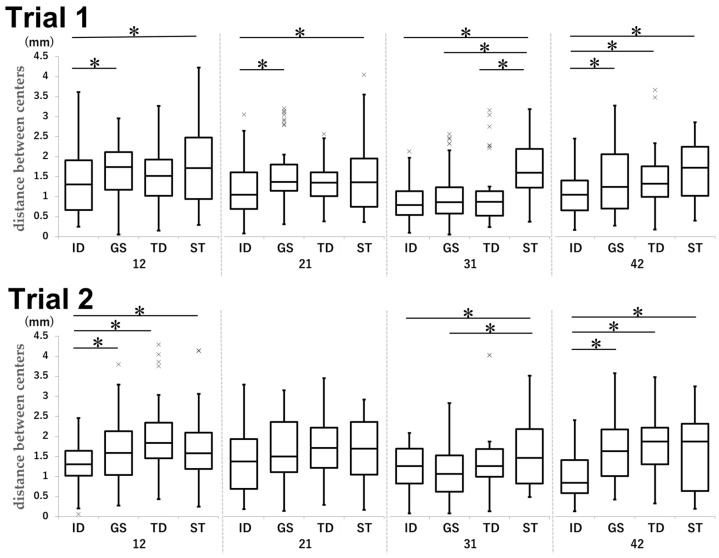
Comparison of the distance between centers in each group and tooth type in Trial 1 and Trial 2. Unit: mm. * *p* < 0.05.

The distance between the centers was significantly smaller in the ID group than in the GS, TD, and ST groups (Figure 5).

For tooth 12 in Trial 1, the ID and GS groups achieved mean distances of 1.33 and 1.62 mm, respectively (*p* = 0.035, d = −0.54), while the ID and ST groups achieved mean distances of 1.33 and 1.77 mm, respectively (*p* < 0.001, d = −0.49). For tooth 21 in Trial 1, the ID group achieved a mean distance of 1.18 mm; in comparison, the GS and ST groups achieved mean distances of 1.59 (*p* < 0.001, d = −0.59) and 1.56 mm (*p* = 0.003, d = −0.48), respectively. For tooth 31 in Trial 1, the mean distances were as follows: ID vs. ST, 0.87 mm vs. 1.69 mm (*p* < 0.001, d = −1.39); GS vs. ST, 1.03 mm vs. 1.69 mm (*p* < 0.001, d = −0.97); and TD vs. ST, 1.07 mm vs. 1.69 mm (*p* < 0.001, d = −0.84). For tooth 42 in Trial 1, the ID group achieved a mean distance of 1.06 mm; in comparison, the GS group achieved a mean distance of 1.47 mm (*p* < 0.001, d = −0.54), the TD group achieved a mean distance of 1.43 mm (*p* = 0.009, d = −0.56), and the ST group achieved a mean distance of 1.64 mm (*p* < 0.001, d = −0.91).

For tooth 12 in Trial 2, the mean distance in the ID group was smaller (1.30 mm) than that in the GS (1.64 mm; *p* = 0.011, d = −0.48), TD (2.04 mm; *p* < 0.001, d = −1.00), and ST (1.70 mm; *p* = 0.003, d = −0.56) groups, respectively. For tooth 31 in Trial 2, the results were as follows: ID vs. ST, 1.23 mm vs. 1.59 mm (*p* = 0.010, d = −0.52) and GS vs. ST, 1.12 mm vs. 1.59 mm (*p* < 0.001, d = −0.64). For tooth 42 in Trial 2, the mean distance in the ID group (1.04 mm) was smaller than that in the GS (1.71 mm; *p* < 0.001, d = −0.95), TD (1.81 mm; *p* < 0.001, d = −1.18), and ST (1.66 mm; *p* < 0.001, d = −0.80), respectively.

**Figure 6:**
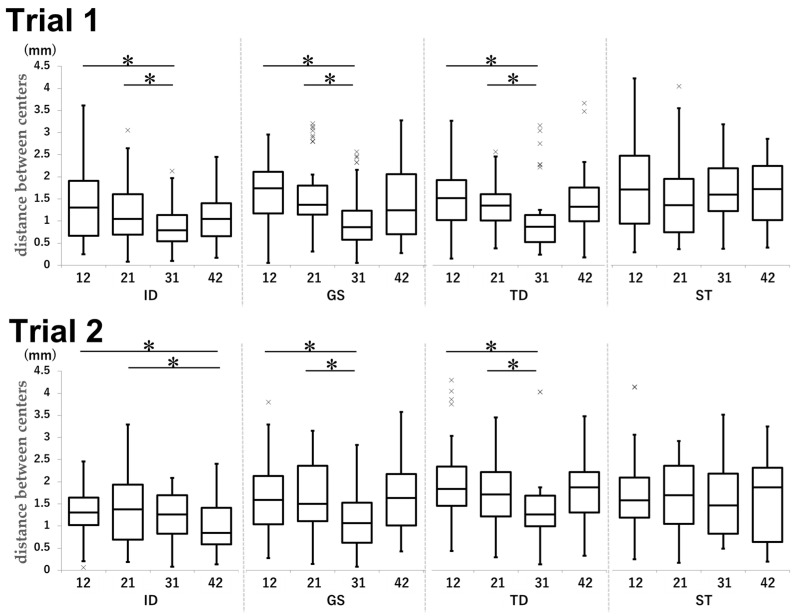
Comparison of the distance between centers by tooth type in each group in Trial 1 and Trial 2. Unit: mm. * *p* < 0.05.

In the case of tooth 31, the distance between the centers was smaller than that of any other maxillary teeth in all groups. This measurement was significantly smaller for 42 than for maxillary teeth in Trial 2 in the ID group (Figure 6).

In Trial 1, the mean distance was 1.30 mm for tooth 12, 1.23 mm for tooth 31, and 1.42 mm for tooth 21 (12 vs. 31: *p* < 0.001, d = 0.71; 21 vs. 31: *p* = 0.0049, d = 0.56) in the ID group. In the GS group, the mean distances were 1.64, 1.12, and 1.64 mm for teeth 12, 31, and 21, respectively (12 vs. 31: *p* < 0.001, d = 0.86; 21 vs. 31: *p* < 0.001, d = 0.79). In the TD group, the mean distances were 2.04 mm for tooth 12, 1.37 mm for tooth 31, and 1.73 mm for tooth 21 (12 vs. 31: *p* < 0.001, d = 0.60; 21 vs. 31: *p* = 0.034, d = 0.39).

In Trial 2, teeth 12, 42, and 21 achieved mean distances of 1.33, 1.06, and 1.18 mm, respectively (12 vs. 42: *p* = 0.040, d = 0.45; 21 vs. 42: *p* < 0.001, d = 0.51), in the ID group. In the GS group, tooth 12 achieved a mean distance of 1.62 mm, tooth 31 achieved a mean distance of 1.03 mm, and tooth 21 achieved a mean distance of 1.59 mm (12 vs. 31: *p* < 0.001, d = 0.68; 21 vs. 31: *p* < 0.001, d = 0.73). The mean distances for teeth 12, 31, and 21 in the TD group were 1.54, 1.07, and 1.32 mm, respectively (12 vs. 31: *p* < 0.001, d = 0.75; 21 vs. 31: *p* = 0.034, d = 0.47).

**Figure 7:**
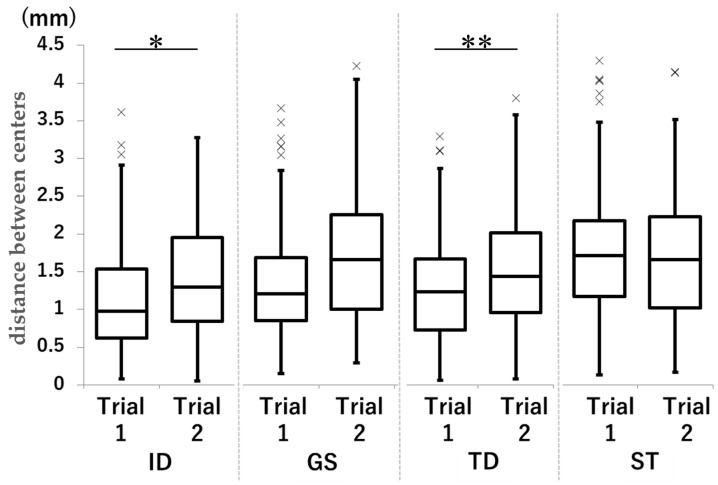
Comparison of the distance between centers in each group in Trial 1 and Trial 2. Unit: mm. * *p* < 0.05. ** *p* < 0.001.

For ID and TD, Trial 1 was smaller than Trial 2 (Figure 7).

In the ID and TD groups, the mean distance was smaller in Trial 1 than in Trial 2 (ID group: 1.11 mm vs. 1.25 mm; *p* = 0.010, d = −0.21; TD group: 1.34 mm vs. 1.74 mm; *p* < 0.001, d = −0.50).

In the less-experienced ST group, the distance between the centers was greater, regardless of DM handling or tooth type. The slightly experienced TD and GS groups appeared relatively similar in their ability to manipulate the mirror with their dominant hand. However, there were differences in the ID results in the trial, manipulating the mirror with the non-dominant hand.

The results for the distance between the centers in each group are shown in Table A1 (Appendix A).

### 3.3. Relative Ratio of BC

**Figure 8:**
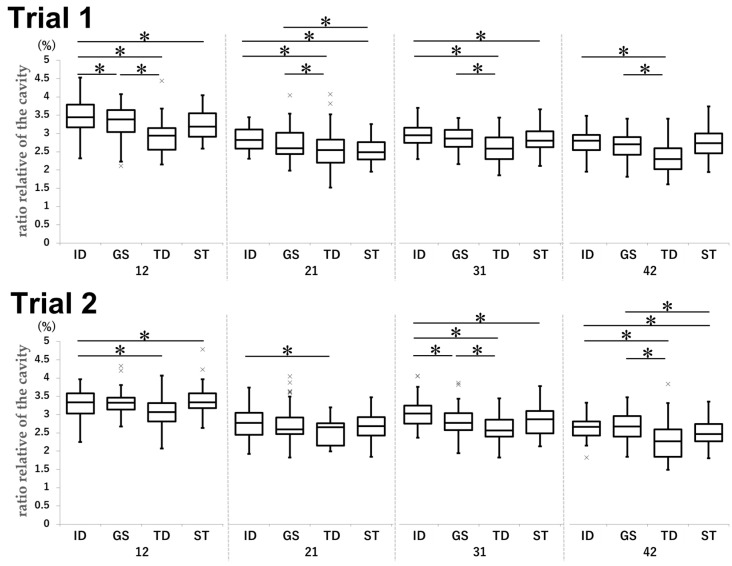
Comparison of the relative ratio of BC by tooth type in each group in Trial 1 and Trial 2. Unit: %. * *p* < 0.05.

The relative BC ratio was significantly higher in the ID group than in the GS, TD, and ST groups. The GS group also showed a significantly higher tendency than the TD and ST groups with less clinical experience (Figure 8).

For tooth 12 in Trial 1, the mean ratio was 3.46% in the ID group, 3.32% in the GS group (ID vs. GS: *p* = 0.028, d = 0.33), 2.93% in the TD group (ID vs. TD: *p* < 0.001, d = 1.18), 3.23% in the ST group (ID vs. ST: *p* < 0.001, d = 0.53), and 3.32% in the GS group (GS vs. TD: *p* < 0.001, d = 0.85). For tooth 21 in Trial 1, the mean ratios were as follows: ID vs. TD, 2.85% vs. 2.56% (*p* < 0.001, d = 0.66); ID vs. ST, 2.85% vs. 2.52% (*p* < 0.001, d = 1.01); GS vs. TD, 2.74% vs. 2.56% (*p* = 0.013, d = 0.36); and GS vs. ST, 2.74% vs. 2.52% (*p* < 0.001, d = 0.56). For tooth 31 in Trial 1, the ID, TD, ST, and GS groups achieved mean ratios of 2.96%, 2.59%, 2.81%, and 2.87%, respectively (ID vs. TD: *p* < 0.001, d = 1.09; ID vs. ST: *p* = 0.035, d = 0.46; GS vs. TD: *p* < 0.001, d = 0.84). For tooth 42 in Trial 1, the mean ratios were larger in the ID group (2.77%) than in the TD group (2.35%; *p* < 0.001, d = 1.16) and in the GS group (2.65%) than in the TD group (2.35%; *p* < 0.001, d = 0.81).

In Trial 2, the following mean ratios were achieved for tooth 12: ID group, 3.28%; TD group, 3.07%; and ST group, 3.38% (ID vs. TD: *p* = 0.0023, d = 0.50; ID vs. ST: *p* < 0.001, d = −0.25). For tooth 21, the mean ratios were 2.78% and 2.51% in the ID and TD groups, respectively (*p* = 0.028, d = 0.65). For tooth 31, the ID group achieved a mean ratio of 3.03%; in comparison, the GS, TD, and ST groups achieved mean ratios of 2.79% (*p* < 0.001, d = 0.57), 2.61% (*p* < 0.001, d = 1.04), and 2.84% (*p* = 0.0037, d = 0.46), respectively. Moreover, the mean ratio in the GS group was larger than that in the TD group (2.79% vs. 2.61%; *p* = 0.013, d = 0.45). For tooth 42, the mean ratios were as follows: ID vs. TD, 2.66% vs. 2.32% (*p* < 0.001, d = 0.81); ID vs. ST, 2.66% vs. 2.51% (*p* = 0.0252, d = 0.48); GS vs. TD, 2.66% vs. 2.32% (*p* < 0.001, d = 0.75); and GS vs. ST, 2.66% vs. 2.51% (*p* = 0.026, d = 0.43).

**Figure 9:**
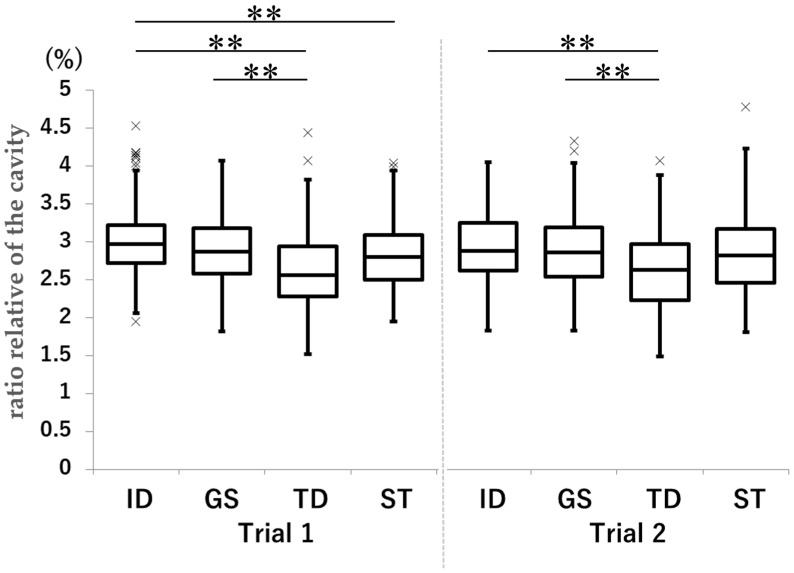
Comparison of the relative ratio of BC in each group in Trial 1 and Trial 2. Unit: %. ** *p* < 0.001.

The value in the ID group was significantly higher in both trials than that in the TD and ST groups, and that in the GS group was significantly higher than that in the TD group in both trials (Figure 9).

The results for Trial 1 were as follows: ID vs. TD, 3.01% vs. 2.61% (*p* < 0.001, d = 0.86); ID vs. ST, 3.01% vs. 2.82% (*p* < 0.001, d = 0.42); and GS vs. TD, 2.89% vs. 2.61% (*p* < 0.001, d = 0.60).

The results for Trial 2 were as follows: ID vs. ST, 2.94% vs. 2.85% (*p* < 0.001, d = −0.64) and GS vs. TD, 2.88% vs. 2.63% (*p* < 0.001, d = 0.50).

The relative ratios of BC in each group are presented in Table A2 (Appendix B).

### 3.4. Ellipticity of BC

**Figure 10:**
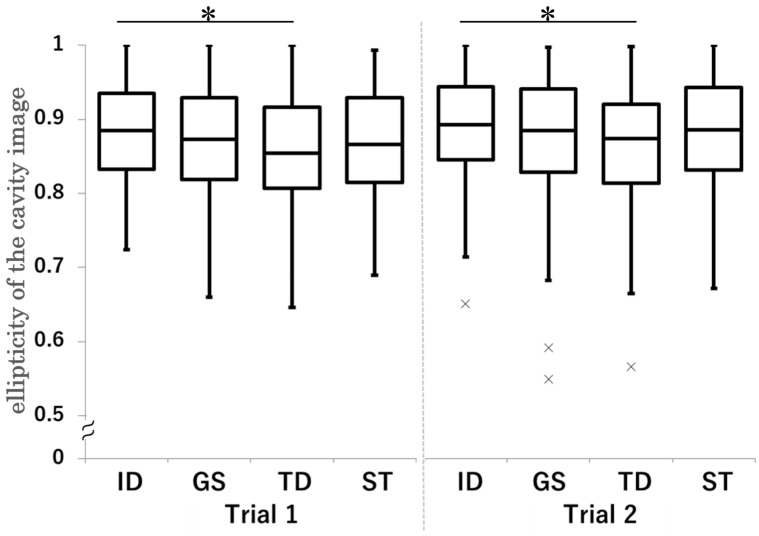
Comparison of the ellipticity of BC in each group in Trial 1 and Trial 2. * *p* < 0.05.

The ellipticity of the BC was higher in the ID group than in the TD group in both trials and was closer to 1 than in the other groups (Figure 10).

In Trial 1, the mean ratio was 0.89 in the ID group and 0.86 in the TD group (*p* < 0.001, d = 0.40).

In Trial 2, the mean ratio was 0.89 in the ID group and 0.86 in the TD group (*p* = 0.0035, d = 0.34).

All results for the ellipticity of BC in each group are shown in Table A3 (Appendix C).

**Figure 11:**
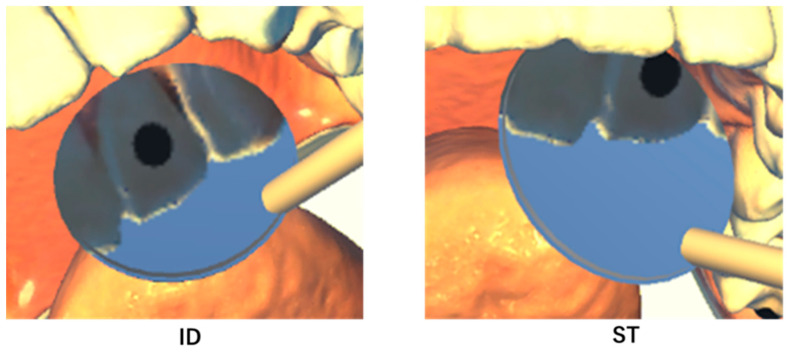
Representative image samples obtained from participants in the ID and ST groups are shown. The ID group positioned the BC significantly closer to the center of the DM than the ST group. In the ID group, the mirror is positioned such that it directly faces the center of the field of view. In the ST group, the mirror is positioned away from the center of the field of view and tilted relative to the direction of gaze.

Figure 11 shows representative examples of the ID and ST results. Regardless of tooth type or differences between Trials 1 and 2, the ID group consistently produced superior results compared to the ST group across all evaluations.

### 3.5. Manipulation Time

**Figure 12:**
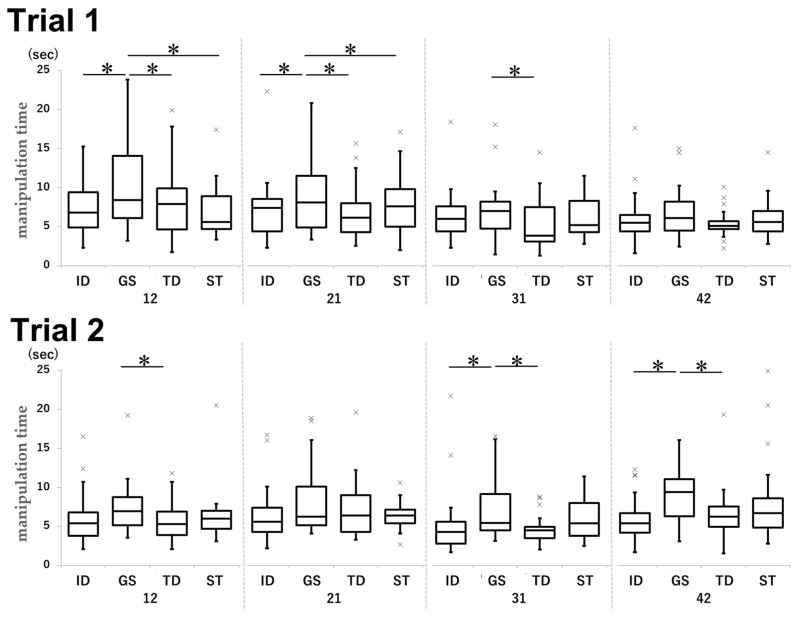
Comparison of the manipulation time by tooth type for each group in Trial 1 and Trial 2. Unit: seconds. * *p* < 0.05.

In both trials, the manipulation time in the GS group was significantly longer than that in the other groups (Figure 12).

In Trial 1, the mean times for tooth 12 were 7.4, 11.3, 8.1, and 7.9 s in the ID, GS, TD, and ST groups, respectively (ID vs. GS: *p* < 0.001, d = −0.67; GS vs. TD: *p* = 0.0014, d = 0.49; GS vs. ST: *p* < 0.001, d = 0.50). For tooth 21, the mean times were 8.3, 11.0, 8.4, and 8.5 s in the ID, GS, TD, and ST groups, respectively (ID vs. GS: p0.0038, d = −0.33; GS vs. TD: *p* < 0.001, d = 0.54; GS vs. ST: *p* = 0.0071, d = 0.38). For tooth 31, the mean time was 8.1 s in the GS group and 6.1 s in the TD group (*p* = 0.023, d = 0.46).

In Trial 2, the mean times achieved in the GS and TD groups were 8.1 and 5.8 s, respectively (*p* = 0.019, d = 0.54). For tooth 31, the ID, GS, and TD groups achieved mean times of 5.3, 7.3, and 4.7 s, respectively (ID vs. GS: *p* = 0.025, d = −0.47; GS vs. TD: *p* = 0.0056, d = 0.74). For tooth 42, the ID, GS, and TD groups achieved mean times of 5.8, 9.6, and 6.7 s, respectively (ID vs. GS: *p* < 0.001, d = −0.89; GS vs. TD: *p* = 0.0011, d = 0.61).

**Figure 13:**
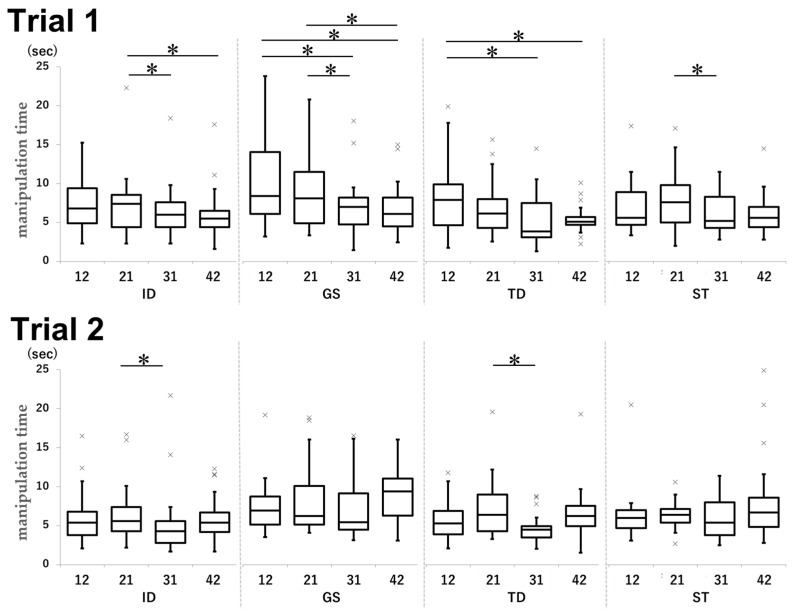
Comparison of the manipulation time for each group by tooth type in Trial 1 and Trial 2. Unit: seconds. * *p* < 0.05.

The manipulation time tended to be shorter for the maxillary sites (12 and 21) than for the mandibular sites (31 and 42) in most groups (Figure 13).

In Trial 1, teeth 21, 31, and 42 in the ID group achieved mean times of 8.3, 6.3, and 6.1 s, respectively (21 vs. 31: *p* = 0.028, d = 0.37; 21 vs. 42: *p* = 0.0097, d = 0.41). In the GS group, teeth 12, 31, 42, and 21 achieved mean times of 11.3, 8.1, 7.7, and 11.0 s, respectively (12 vs. 31: *p* < 0.001, d = 0.37; 12 vs. 42: *p* < 0.001, d = 0.45; 21 vs. 31: *p* < 0.001, d = 0.45; 21 vs. 42: *p* < 0.001, d = 0.55). In the TD group, the results were as follows: 12 vs. 31, 8.1 s vs. 5.6 s (*p* = 0.0030, d = 0.59) and 12 vs. 42, 8.1 s vs. 5.5 s (*p* = 0.0016, d = 0.70). In the ST group, tooth 21 group achieved a mean time of 8.4 s while tooth 31 achieved a mean time of 6.1 s (*p* = 0.0097, d = 0.68).

In Trial 2, the mean times were 7.4 s for tooth 21 and 5.3 s for tooth 31 in the ID group (*p* = 0.0047, d = 0.38), while they were 8.3 s for tooth 21 and 4.7 s for tooth 31 in the TD group (*p* < 0.001, d = 0.80).

**Figure 14:**
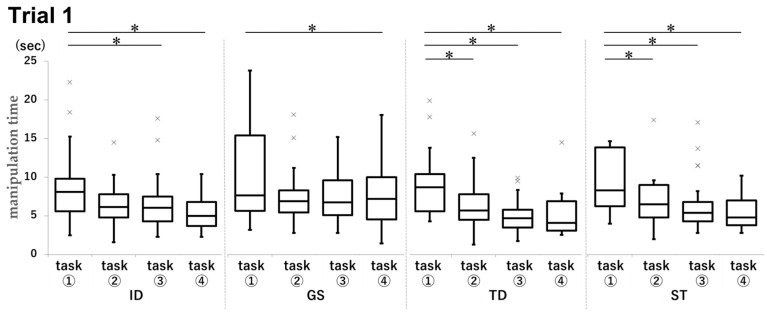
Comparison of manipulation times by task for each group in Trial 1. Unit: seconds. * *p* < 0.05.

In Trial 1, the manipulation time for task ① was significantly longer than that for the other tasks across all groups (Figure 14).

In Trial 1, the mean manipulation time for task ① in the ID group was 8.6 s, compared to 6.6 s for task ③ (*p* = 0.0246, d = 0.50) and 5.4 s for task ④ (*p* < 0.001, d = 0.93). In the GS group, the mean time was 10.8 s for task ① and 8.9 s for task ④ (*p* = 0.046, d = 0.25). In the TD group, the task ① group achieved a mean time of 9.2 s, compared to 6.7 s in the task ② group (*p* = 0.0018, d = 0.63), 5.0 s in the task ③ group (*p* < 0.001), d = 1.19), and 5.4 s in the task ④ group (*p* < 0.001, d = 1.03). In the ST group, the task ① group achieved a mean time of 10.4 s, compared to 7.1 s in the task ② group (*p* < 0.001, d = 0.62), 6.6 s in the task ③ group (*p* < 0.001, d = 0.68), and 5.5 s in the task ④ group (*p* < 0.001, d = 0.95).

All data for the manipulation time in each group are shown in Table A4 (Appendix D).

### 3.6. Questionnaire

**Figure 15:**
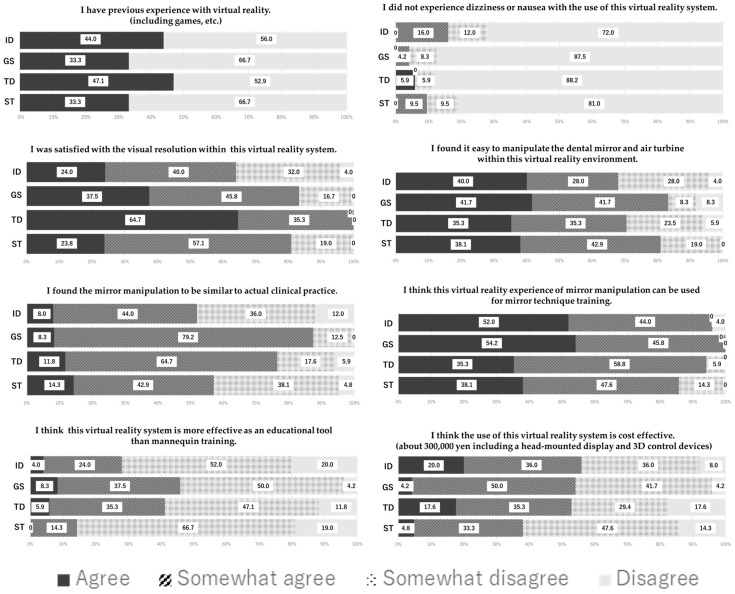
Results of the questionnaire.

All participants completed the questionnaire. For question 1, less than half the participants mentioned having prior VR experience in all groups. For question 2, 80–90% participants were able to complete the experiment without cybersickness. Regarding questions 3 to 5, over half the participants in all groups provided positive responses concerning resolution and operability, with slightly more negative opinions observed in the ID group. For question 6, over 80% participants in all groups were affirmative regarding the effectiveness of the system in MT education. Responses to question 7 indicated that this VR system was superior than manikin-based training for MT. Regarding question 8, opinions on the cost of this system were evenly distributed as favorable and unfavorable. The detailed survey results are shown in Figure 15.

## 4. Discussion

It is necessary to position the object quickly at the center of the mirror surface to maximize its size and minimize distortion during dental procedures with the dental mirror. The VR system developed in this study enabled the sharing of mirror images and the objective evaluation of objects reflected in the DM.

### 4.1. Distance Between Centers

The distance between the centers indicates that the object is reflected closer to the center of the mirror and is considered an indicator of visibility. This study revealed that the distance between the centers in the ID group was close to zero. As shown in Figure 10, regardless of the trial type or target site, the ST group with less clinical experience exhibited a larger intra-center distance. Furthermore, for results involving the nondominant hand, there were clear differences between the slightly more experienced GS and TD groups and the ID group. These findings suggest that the accumulation of clinical experience is related to MT skill. Similarly to previous reports [28], the fact that Trial 1 showed significantly smaller values in both the ID and TD groups suggests that even after acquiring a certain level of experience, the dexterity of the dominant hand remained superior in terms of precise movements.

### 4.2. Relative Ratio of BC

The relative ratio of BC indicates the size of the objects visible on the mirror surface and affects the detailed examination. The ID group demonstrated higher values than the other groups. The higher scores observed in the ID and GS groups than in the TD group suggest that they had acquired effective techniques for using dental mirrors through experience and had performed appropriate manipulation. The significantly higher values observed for the maxillary teeth compared to the mandibular teeth were associated with limitations of the practical position. Additionally, the lack of an object intrusion prevention function in this VR system made it difficult for participants to judge the distances.

### 4.3. Ellipticity of BC

The ellipticity of the BC in the ID group was close to 1, indicating that the BC was projected as a perfect circle. It was suggested that the participants in the ID group had sufficient skills for proper manipulation of the DM, even in the restricted clinical condition, and the result was deemed applicable to evaluate the angle of the dental mirror or practical position of the operator in the future.

### 4.4. Manipulation Time

Manipulation time serves as an indicator of the smoothness of dental mirror manipulation and the efficiency of dental treatment. Although all participants took significantly longer time in the initial task ①, subsequent tasks showed no significant differences. These results indicate that the participants did not require much time to become accustomed to the VR environment or its controls. The lack of significant differences between Trials 1 and 2 could be attributed to the relatively simple content of the trials.

### 4.5. Questionnaire

In this experiment, the short duration of stay in the VR environment was considered the reason for the low incidence of cybersickness [29,30,31]. The favorable opinions regarding the application of this system to MT education were related to resolution and operability. Questions 3 to 5 received numerous favorable responses; this suggested that educational tools do not necessarily require a pursuit of realism. The responses to questions 7 and 8 were considered related to the initial investment required for VR equipment.

### 4.6. Participants

This study involved participants with varying levels of clinical experience, enabling the quantitative assessment of skill differences between the ID group participants routinely utilizing MT across multiple fields and less-experienced participants in the GS, TD, and ST groups. The GS group showed similar results for the distance between centers, the relative ratio of BC, and the ellipticity of BC as the ID group but had a longer manipulation time. This result is related to dental residency training and graduate school curricula in Japan and was thought to be associated with graduate students in the research environment having relatively less time spent in clinical practice.

### 4.7. Dental Mirror VR System and Learning Effects

Although this study could not rigorously verify the relationship between skill assessment using the VR system and clinical performance, the results for the ID group were clearly superior to those for the other groups. Therefore, it was considered meaningful to evaluate MT skills using the items and system established in this study. Furthermore, the results differed significantly between task ① and subsequent tasks; this suggested that differences in learning effects within the same trial were greater than those between Trials 1 and 2.

### 4.8. Future Outlook

Comparing the hapTEL simulator Simodont with our system, we found that the latter has the disadvantage of the lack of haptic feedback; however, it possesses a significant advantage in terms of enabling operators to share their field of view with others. The VR system includes a function that outputs the user’s field of view to a monitor and records the manipulations performed within the VR environment. Utilizing these functions enables the provision of immediate feedback based on objective evaluation as well as the simulated experience of demonstrations performed by supervising dentists. The survey results also revealed numerous positive opinions regarding the usefulness of this system as an educational tool. Reliving experiences through VR has been reported to provide greater realism and immersion compared to two-dimensional monitor images [18,32,33,34,35], and the use of VR devices has significantly expanded in recent years for education on dental clinical skills [36,37,38,39]. Through continuous refinement, the VR system has the potential to evolve into an unprecedented educational tool for MT.

### 4.9. Limitations

The construction of the reflected image of the VR mirror required complex settings. With future application to the molar region in mind, we initially focused on the anterior region to achieve the simplest conditions. However, in clinical practice, there are numerous areas that cannot be evaluated without the use of MT, such as the distal surfaces of the most posterior molars. Furthermore, as mentioned earlier, this VR system lacks an object penetration prevention function. These represent the limitations of this study and are considered future challenges.

## 5. Conclusions

The newly developed VR system enables the objective evaluation of MT skills, and our findings suggest that MT is influenced by clinical experience. This VR platform can serve not only for assessment but also for personalized feedback and curriculum integration. By utilizing shared mirror images and recording functions, this VR system has the potential to become an effective tool for MT education. In order to achieve effective education using this VR system, further studies with larger samples and more complex clinical tasks are needed.

## Figures and Tables

**Table 1:** Characteristics of participants.

		ID	GS	TD	ST
age (years)	range	33–60	25–34	24–32	23–27
	average	43.8 ± 8.3	27.9 ± 2.1	25.2 ± 1.9	23.8 ± 1.1
sex (people)	men	15	12	9	8
	women	10	12	8	13
clinical experience		9 to 36 years	1 to 4 years	3 months of clinical training	6 months since the start of clinical practice
dominant hand (people)	right	25	24	17	18
	left	0	0	0	3

## Data Availability

The data supporting the findings of this study are available from the corresponding author upon request.

## Supplementary Materials

The following supporting information can be downloaded at https://www.mdpi.com/article/10.3390/dj13120566/s1, Figure S1: Calculation Methods for Data Analysis.

## Abbreviations

The following abbreviations are used in this manuscript:

VR	Virtual reality
MT	Mirror technique
HMD	Head-mounted display
DM	Virtual dental mirror
TH	Air turbine handpiece
BC	Black caries lesion
ID	Instructor dentists
GS	Graduate student
TD	Trainee dentist
ST	Student

## Appendix A

**Table A1:** Distance between centers.

		ID	GS	TD	ST
	Tooth	Median (IQR)	Median (IQR)	Median (IQR)	Median (IQR)
Trial 1	12	1.30 (0.67–1.91)	1.74 (1.17–2.12)	1.52(1.02–1.93)	1.72 (0.94–2.48)
	21	1.05 (0.69–1.61)	1.36 (1.15–1.81)	1.35 (1.01–1.61)	1.36 (0.75–1.96)
	31	0.79 (0.55–1.14)	0.86 (0.57–1.24)	0.87 (0.52–1.14)	1.59 (1.23–2.19)
	42	1.04 (0.66–1.40)	1.24 (0.71–2.06)	1.32 (1.00–1.76)	1.72 (1.02–2.25)
Trial 2	12	1.31 (1.02–1.64)	1.59 (1.04–2.13)	1.84 (1.46–2.34)	1.58 (1.19–2.10)
	21	1.38 (0.69–1.93)	1.50 (1.11–2.36)	1.71 (1.22–2.22)	1.69 (1.04–2.36)
	31	1.26 (0.83–1.69)	1.06 (0.62–1.53)	1.26 (1.00–1.69)	1.47 (0.83–2.18)
	42	0.84 (0.59–1.41)	1.63 (1.01–2.17)	1.88 (1.31–2.22)	1.88 (0.64–2.32)

Median (first quartile–third quartile). Unit: mm. ID, instructor dentist; GS, graduate student; TD, trainee dentists; ST, student.

## Appendix B

**Table A2:** Relative ratio of black caries lesion.

		ID	GS	TD	ST
	Tooth	Median (IQR)	Median (IQR)	Median (IQR)	Median (IQR)
Trial 1	12	3.44 (3.16–3.78)	3.38 (3.04–3.64)	2.94 (2.56–3.14)	3.19 (2.91–3.56)
	21	2.83 (2.59–3.11)	2.60 (2.43–3.02)	2.55 (2.20–2.83)	2.48 (2.29–2.77)
	31	2.95 (2.74–3.16)	2.86 (2.63–3.10)	2.58 (2.30–2.89)	2.80 (2.63–3.06)
	42	2.81 (2.55–2.96)	2.71 (2.42–2.90)	2.30 (2.02–2.59)	2.73 (2.46–3.00)
Trial 2	12	3.33 (3.03–3.58)	3.32 (3.14–3.46)	3.06 (2.81–3.32)	3.34 (3.17–3.58)
	21	2.77 (2.45–3.05)	2.60 (2.47–2.92)	2.65 (2.15–2.76)	2.68 (2.43–2.93)
	31	3.03 (2.75–3.25)	2.78 (2.57–3.04)	2.57 (2.40–2.86)	2.87 (2.48–3.10)
	42	2.66 (2.43–2.81)	2.67 (2.39–2.96)	2.27 (1.84–2.60)	2.47 (2.27–2.74)

Median (first quartile–third quartile). Unit: %. ID, instructor dentist; GS, graduate student; TD, trainee dentists; ST, student.

## Appendix C

**Table A3:** Ellipticity of black caries lesion.

		ID	GS	TD	ST
	Tooth	Median (IQR)	Median (IQR)	Median (IQR)	Median (IQR)
Trial 1	12	0.89 (0.82–0.95)	0.88 (0.84–0.94)	0.86 (0.81–0.91)	0.87 (0.84–0.93)
	21	0.86 (0.81–0.90)	0.85 (0.80–0.87)	0.82 (0.81–0.86)	0.85 (0.79–0.89)
	31	0.91 (0.87–0.94)	0.92 (0.84–0.95)	0.92 (0.84–0.95)	0.88 (0.81–0.96)
	42	0.89 (0.84–0.95)	0.88 (0.81–0.95)	0.85 (0.80–0.89)	0.87 (0.83–0.94)
Trial 2	12	0.89 (0.84–0.95)	0.88 (0.83–0.94)	0.87 (0.82–0.90)	0.89 (0.84–0.94)
	21	0.84 (0.78–0.90)	0.80 (0.78–0.84)	0.79 (0.72–0.87)	0.83 (0.78–0.88)
	31	0.93 (0.88–0.96)	0.91 (0.87–0.95)	0.91 (0.85–0.95)	0.94 (0.88–0.97)
	42	0.90 (0.87–0.96)	0.92 (0.87–0.95)	0.89 (0.85–0.93)	0.90 (0.83–0.93)

Median (first quartile–third quartile). ID, instructor dentist; GS, graduate student; TD, trainee dentists; ST, student.

## Appendix D

**Table A4:** Manipulation time.

a					
		**ID**	**GS**	**TD**	**ST**
	**tooth**	**Median (IQR)**	**Median (IQR)**	**Median (IQR)**	**Median (IQR)**
Trial1	12	6.8 (4.9–9.4)	8.4 (6.1–14.1)	7.9 (4.6–9.9)	5.6 (4.7–8.9)
	21	7.4 (4.4–8.5)	8.1 (4.9–11.5)	6.2 (4.3–8.0)	7.6 (5.0–9.8)
	31	6.0 (4.4–7.6)	7.0 (4.7–8.2)	3.8 (3.1–7.5)	5.2 (4.3–8.3)
	42	5.5 (4.4–6.5)	6.1 (4.5–8.2)	5.1 (4.7–5.7)	5.6 (4.4–7.0)
Trial2	12	5.4 (3.8–6.8)	7.0 (5.2–8.7)	5.3 (3.9–6.9)	6.0 (4.7–7.0)
	21	5.6 (4.3–7.4)	6.2 (5.1–10.1)	6.4 (4.3–9.0)	6.4 (5.4–7.2)
	31	4.3 (2.8–5.6)	5.4 (4.5–9.1)	4.5 (3.5–5.0)	5.4 (3.8–8.0)
	42	5.4 (4.2–6.7)	9.4 (6.3–11.1)	6.2 (4.9–7.5)	6.7 (4.8–8.6)
b					
		**ID**	**GS**	**TD**	**ST**
	**Task**	**Median (IQR)**	**Median (IQR)**	**Median (IQR)**	**Median (IQR)**
Trial1	①	8.1 (5.6–9.8)	7.6 (5.7–15.4)	8.7 (5.6–10.4)	8.3 (6.2–13.9)
	②	6.2 (4.8–7.8)	6.9 (5.5–8.3)	5.7 (4.5–7.8)	6.5 (4.8–9.0)
	③	6.0 (4.3–7.5)	6.7 (5.1–9.6)	4.7 (3.5–5.8)	5.4 (4.3–6.8)
	④	5.0 (3.7–6.8)	7.2 (4.5–10.0)	4.1 (3.1–6.9)	4.8 (3.8–7.0)
Trial2	①	5.5 (4.3–6.6)	6.6 (4.9–9.2)	4.9 (4.3–6.2)	6.6 (5.4–8.0)
	②	5.4 (3.5–7.4)	6.3 (5.3–9.9)	5.8 (4.2–7.1)	6.5 (4.8–7.2)
	③	5.3 (4.0–6.3)	6.4 (4.5–11.3)	5.6 (4.5–8.6)	5.6 (4.6–8.3)
	④	4.5 (3.3–6.0)	7.0 (5.6–10.1)	5.9 (3.5–7.9)	5.4 (4.9–6.9)

Median (first quartile–third quartile). Unit: seconds. (a) Results according to tooth type in Trials 1 and 2. (b) Task results of Trials 1 and 2. ID, instructor dentist; GS, graduate student; TD, trainee dentists; ST, student.
