# Supplementary material for: New Virtual Reality Educational Tool for Evaluating Dental Mirror Technique Skills: A Pilot Study
Source: Dent J (Basel). 2025 Dec 1;13(12):566. doi: 10.3390/dj13120566 (PMC12731970; doi:10.3390/dj13120566)
Supplement: Supplementary file 1 [file dentistry-13-00566-s001.zip › Figure S1.pdf]

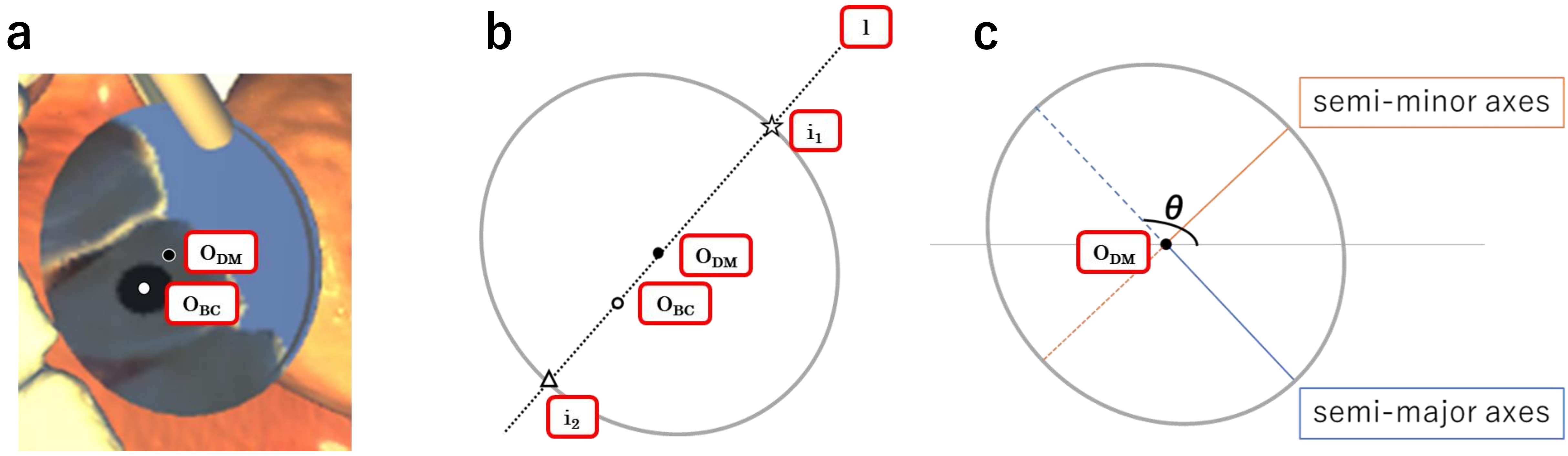

d The distance between the center of the DM and BC mirror image (distance between centers) d_{VR} was calculated geometrically using coordinate, semi-major and semi-minor axes, angle, etc. obtained from ImageJ.

Since the distance between centers d_{VR} (pixels) is the distance between points O_{DM} and O_{BC},

$$d_{VR} = \sqrt{(X_{BC} - X_{DM})^2 + (Y_{BC} - Y_{DM})^2}$$

Considering the tilt and distortion of the DM ellipse, convert the units from pixels to mm. Since the line l passes through O_{DM}, which is the center of the DM and O_{BC}, the distance d_i between l and the ellipse's intersection points $i_1(X_{i1}, Y_{i1})$ and $i_2(X_{i2}, Y_{i2})$, the DM diameter, equals 20.0 mm. Therefore, the distance between centers d_R(mm) is calculated using d_{VR} and d_i as follows:

$$\frac{d_R}{d_{VR}} = \frac{20}{d_i}$$

That is to say,

$$d_R = 20 \times \frac{d_{VR}}{d_i} \ldots (1)$$

Here, we derive the equation of the DM ellipse. The equation of an ellipse centered at the origin is as follows: $$\frac{x^2}{a^2} + \frac{y^2}{b^2} = 1 \ldots (2)$$

The point $a(X_a, Y_a)$ on the ellipse of the DM is the point rotated by θ about the origin and translated by O_{DM} (X_{DM}, Y_{DM}). Using linear transformations, these can be expressed as follows:

$$X_a = (x - X_{DM})\cos\theta + (y - Y_{DM})\sin\theta \ldots (3)$$
$$Y_a = -(x - X_{DM})\sin\theta + (y - Y_{DM})\cos\theta \ldots (4)$$

The equation of the ellipse of the DM is obtained by substituting (3) and (4) into the x and y variables of equation (2), respectively.

On the other hand, the equation of line l is expressed as follows:

$$l : y - Y_{DM} = \frac{Y_{DM} - Y_{BC}}{X_{DM} - X_{BC}}(x - X_{DM}) \ldots (5)$$

Therefore, by solving the simultaneous equations of the elliptic equation of the DM and the equation of line l, the intersection points $i_1(X_{i1}, Y_{i1})$ and $i_2(X_{i2}, Y_{i2})$ of the ellipse can be determined. The distance d_i between the intersection points is expressed as follows:

$$d_i = \sqrt{(X_{i1} - X_{i2})^2 + (Y_{i1} - Y_{i2})^2}$$

Substituting the obtained d_{VR} and d_i into equation (1) allows the distance between centers d_R to be determined.

e The area of the DM ($Area_{DM}$) and the area of the BC ($Area_{BC}$) were measured, and the relative ratio of BC (%) was calculated using the following formula:

$$R = \frac{Area_{BC}}{Area_{DM}} \times 100$$

f The semi-major axis (a_{BC}) and semi-minor axis(b_{BC}) of the ellipse of BC were measured, and the ellipticity of BC k was calculated using the following formula:

$$k = \frac{b_{BC}}{a_{BC}}$$

Supplementary Figure S1: Calculation methods for data analysis

a) ●: Center coordinates of DM, $O_{DM}(X_{DM}, Y_{DM})$, ○: center coordinates of BC, $O_{BC}(X_{BC}, Y_{BC})$. b) ●: Center coordinates of DM, $O_{DM}(X_{DM}, Y_{DM})$, ○: center coordinates of BC, O_{BC} (X_{BC}, Y_{BC}), ---- : line l passes through points O_{DM} and O_{BC}, ☆/△: points i_1 and i_2 are the intersections of line l and the ellipse of the mirror frame. c) ●: Center coordinates of DM, $O_{DM}(X_{DM}, Y_{DM})$, θ: x-coordinate and ellipse slope. d) Calculation method for the distance between the center of the DM and BC mirror image. e) Calculation method for the relative ratio of BC mirror image on the DM surface. f) Calculation method for the ellipticity of BC mirror image on the DM.
